# Thermography of cannabis extract vaporization cartridge heating coils in temperature- and voltage-controlled systems during a simulated human puff

**DOI:** 10.1371/journal.pone.0262265

**Published:** 2022-01-26

**Authors:** Michael A. Oar, Cynthia H. Savage, Echoleah S. Rufer, Richard P. Rucker, Jesse A. Guzman

**Affiliations:** 1 Product Integrity, PAX Labs, Inc., San Francisco, California, United States of America; 2 Biocompatibility and Toxicology, PAX Labs, Inc., San Francisco, California, United States of America; Tongji University, CHINA

## Abstract

Vaporized cannabis is believed to be safer than smoking, but when heated to excessive temperatures nearing combustion (>900 °C) harmful byproducts may form. While some cannabis extract vaporizers operate well below these high temperatures, heating coil temperatures obtained during actual use are frequently not reported and many operate at high temperatures. We report on two major objectives: 1) development of an infrared thermography method to measure heating coil temperatures in cannabis extract vaporizers during a simulated puff and 2) a comparison of temperature- to voltage- controlled cannabis extract vaporization systems during a puff. Infrared thermography was used to measure heating coil temperatures in one temperature-controlled and two voltage-controlled systems. The cartridges were modified for direct line-of-sight on the heating coils, the wick and coils were saturated with cannabis extract, and fixtures were developed to force two liters per minute air flow past the coils for the full duration of the puff allowed by the device. The voltage-controlled systems produced higher temperatures with greater variability than the temperature-controlled system. At the highest temperature setting (420 °C) the temperature-controlled system reached an average heating coil temperature of 420 ± 9.5 °C whereas the 4.0V setting on the variable voltage system reached an average temperature of 543 ± 95.9 °C and the single voltage (3.2V) system an average of 450 ± 60.8 °C. The average temperature at the lowest setting (270 °C) on the temperature-controlled system was 246 ± 5.1 °C and the variable voltage system (2.4V) was 443 ± 56.1 °C. Voltage alone was a poor indicator of coil temperature and only the temperature-controlled system consistently maintained temperatures less than 400 °C for the full puff duration. These lower temperatures could reduce the likelihood of harmful thermal degradation products and thus may reduce potential health risk to consumers when vaporizing cannabis extracts.

## Introduction

Over the last decade, vaporization of cannabis has gained popularity due to its convenience and reported reduced health risk relative to cannabis smoking/combustion methods [1–5]. Vaporization of cannabis flower and extracts typically utilizes temperatures much lower than those required for smoking cannabis joints. The concentration of harmful thermal degradation products emitted during vaporization is greatly reduced relative to smoked cannabis because of the lower temperatures employed by vaporization [6, 7]. Similar trends have also been observed for “heat not burn” tobacco products and e-cigarettes when compared to cigarette smoking [8–10]. However, the 2019–2020 e-cigarette or vaping product use associated lung injury (EVALI) outbreak was associated with illegally purchased devices containing vitamin E acetate, possibly associated with chemical reactions with internal cartridge components and high (>700 °C) coil temperatures [11–14]. In addition, temperature may also affect particle size and lung deposition which could have significant impacts on effect of the product for the consumer [15]. Therefore, accurate characterization of coil temperatures from cannabis extract vaporizers may be important in determining potential health effects on consumers. This study measured heating coil temperatures from several cannabis extract vaporization systems during a simulated human puff to inform on potential health effects which are hypothesized to differ from electronic nicotine delivery systems (ENDS).

**Fig 1:**
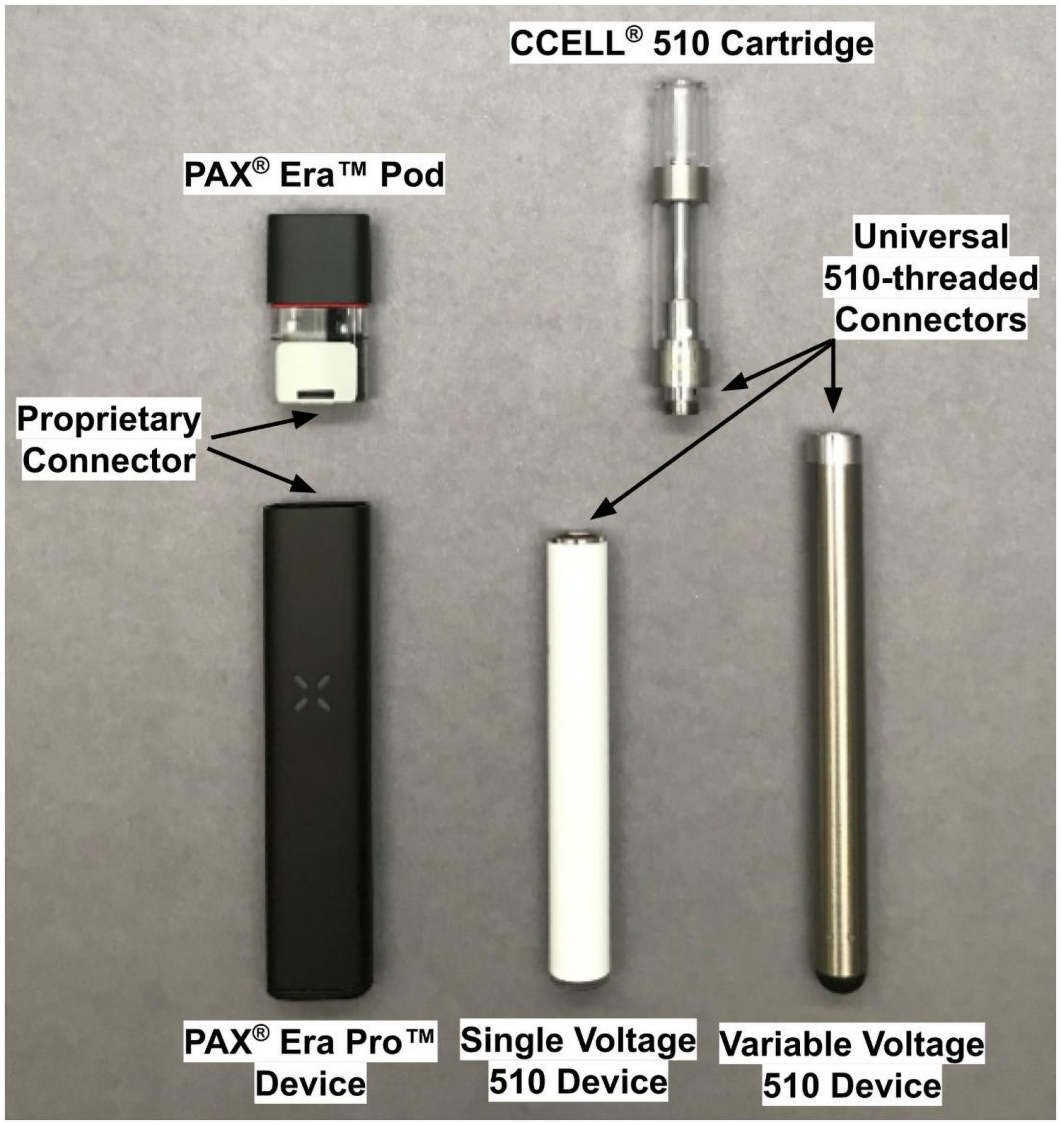
Vaporization systems used in this study.

Cannabis extract vaporization systems often utilize a disposable cartridge (“cart” for short) or pod connected to a rechargeable battery-powered device (also called a vape pen, battery, or device). Cartridges and devices are sold as either a proprietary system, which only function when paired to specific units within the ecosystem or as a universal system, of which 510-threaded products are the most common. All cartridges and devices in the 510 ecosystem are connected to one another using a universal mating 510 thread design (Fig 1). This interchangeable nature of 510-systems suggests a likelihood for variable temperatures.

The cannabis extract vaporization cartridges examined contain an internal metallic heating coil filament that generates the heat required to produce aerosol when the consumer puffs on the device. Device settings used to control temperature may often be in watts, volts, or degrees (°C or °F). In general, setting the device to a higher voltage or wattage setting increases the coil temperature, however it is oftentimes not clear what temperature that setting equates to. Unfortunately, most devices only provide settings for power output rather than temperature [11]. In addition, the same voltage setting on two devices may not generate the same heating coil temperature in all systems [16, 17]. Precise measurement of the cartridge heating coil during human-use conditions is difficult because the coil is physically embedded inside of the cartridge. A few 510 devices report coil temperature estimates, but these values are inherently quite variable because of the wide range of 510 cartridges that are manufactured using a variety of coil metal compositions and coil resistance values. To our knowledge, direct and accurate measurement of cannabis extract pod/cartridge coil temperatures has not been reported.

Vaporizer heating coil analysis has been performed on ENDS [18–20]. It was found that temperature can vary widely along the length of the heating coil due to the nature of resistive heating, heat sinking from the electrical leads, heat transfer to the surrounding elements, air movement past the coil, the presence of vaporizable liquids, and wick desaturation during a puff [18–20]. ENDS heating coil temperatures were previously measured using thermocouples [18] or infrared (IR) thermography but have not been measured on cannabis extract vaporizers [19, 20]. Thermocouple analysis uses a metallic probe to contact the heating coil and only measures the temperatures at a single point along the coil. IR thermography is a thermal imaging technique which uses infrared light radiated from a heat source to measure its temperature. Thermography is preferred relative to thermocouple analysis because thousands of non-contact measurements can be made over a much larger area along the length of the coil [19, 20]. A metallic thermocouple contact probe can also draw heat from a small thermal mass object like the vaporizer coils in this study, which leads to inaccurate temperature measurements. Thermography also allows time- and spatially-resolved temperature measurements along the length of the coil throughout the entire puff event.

Resistive heating coils are frequently made from nichrome metal because this alloy is capable of rapid heating and its temperature can be determined through resistance measurements and the use of the Temperature Coefficient of Resistivity (TCR) calculation [18]. TCR describes the change in coil resistance versus temperature and is the basis for temperature monitoring and control. Closed-loop temperature control is achieved by monitoring the resistance of the nichrome heating during a puff event. Since the coil resistance is directly related to its temperature through TCR, the vaporization device can apply power to the coil until the appropriate resistance is measured. In order to rapidly reach the temperature set point and continuously maintain the desired temperature, a proportion-integral-derivative (PID) control loop mechanism is employed. This PID control mechanism automatically responds and corrects the coil temperature using the TCR relationship. In contrast to the more complex and dynamic management used in temperature-controlled devices, the main feasible alternative that is employed is the static voltage-controlled mechanism. Voltage-controlled devices apply a single voltage across the coils independent of 510 cartridge style, heating coil composition, and temperature without a feedback loop to control temperature.

Accurate measurement of cannabis extract vaporizer coil temperatures is important in determining potential health risk to consumers. As such, in this study we aimed to expand the data available in the published literature by: 1) developing a test method to accurately measure the heating coil temperatures during simulated human draw events and 2) conducting an analysis of the heating coil temperatures of a closed-loop temperature-controlled system versus the open-loop voltage-controlled 510 systems. This information will be important in better understanding the operating temperatures of these devices which will help to identify potential thermal degradation products and health risks from cannabis vaporization products.

## Materials and methods

### Cannabis extract and vaporization systems

Three handheld cannabis vaporization systems—a single temperature-controlled (TC) and two voltage-controlled—were characterized in this study (Fig 1). The objective was to compare a temperature-controlled system to the most common alternative, 510-compatible batteries and CCELL^®^ 510 cartridges. The PAX^®^ Era Pro^™^ is one of the only devices that allows the consumer to set temperatures (of the coil) in one degree increments from 220 to 420 °C (430–790 °F) via PAX’s web or mobile application. The PAX^®^ Era Pro^™^ is a common product in a top price tier. The variable voltage (VV510) device allows the consumer to set voltages at 2.4V (low), 3.2V (medium), and 4.0V (high). The single voltage 510 (SV510) device is permanently set to 3.2V. Details of the devices used are shown in Table 1. These popular devices were chosen to span the range of devices from different brands available with the VV510 device being in the higher price range and the SV510 device being one of the more inexpensive devices that might be offered as giveaway with purchase of a 510 cartridge. All three devices are draw-activated.

**Table 1:** Vaporization systems tested.

Code	Device	Cartridge	Coil	Puff length	Temperature and Voltage Settings
TC	PAX^®^ Era Pro^™^ temperature-controlled device	0.5 g PAX^®^ Era^™^ pod	4-turn Nichrome	15 sec	Lowest and highest default temperature setting (270 and 420 °C)
VV510	Variable voltage 510 device from Brand A	1 g 510-threaded CCELL^®^ M6T10 cartridge	3-turn Nichrome	8 sec for 2.4 V and 4 sec for 4.0 V	Lowest and highest voltage setting (2.4 and 4.0 V)
SV510	Single voltage 510 device from Brand B			10 sec	3.2 V

All CCELL^®^ 510 cartridges used in this study were 1-gram cartridges that could be screwed into the VV510 and SV510 devices, purchased from a dispensary. The main components of the 510 cartridges included a plastic tank and a ceramic-encased heating coil. PAX^®^ Era^™^ pods used in this study were those commercially available in the California regulated cannabis market, comprised of a plastic tank and a heating coil wrapped around an amorphous silica wick.

The cannabis extract used for all measurements in this study was a commercially available Maui Wowie strain containing 87% tetrahydrocannabinol (THC) purchased from a state-regulated adult use cannabis dispensary in California. For the temperature-controlled (TC) system, PAX^®^ Era^™^ pods were filled with the cannabis extract and vaporized using a PAX^®^ Era Pro^™^ device (firmware version 4.2.3). For the voltage-controlled systems, 510-threaded cartridges were filled with the same cannabis extract and were vaporized using VV510 and SV510 devices.

### Preparation of cartridges and pods for temperature measurement

**Fig 2:**
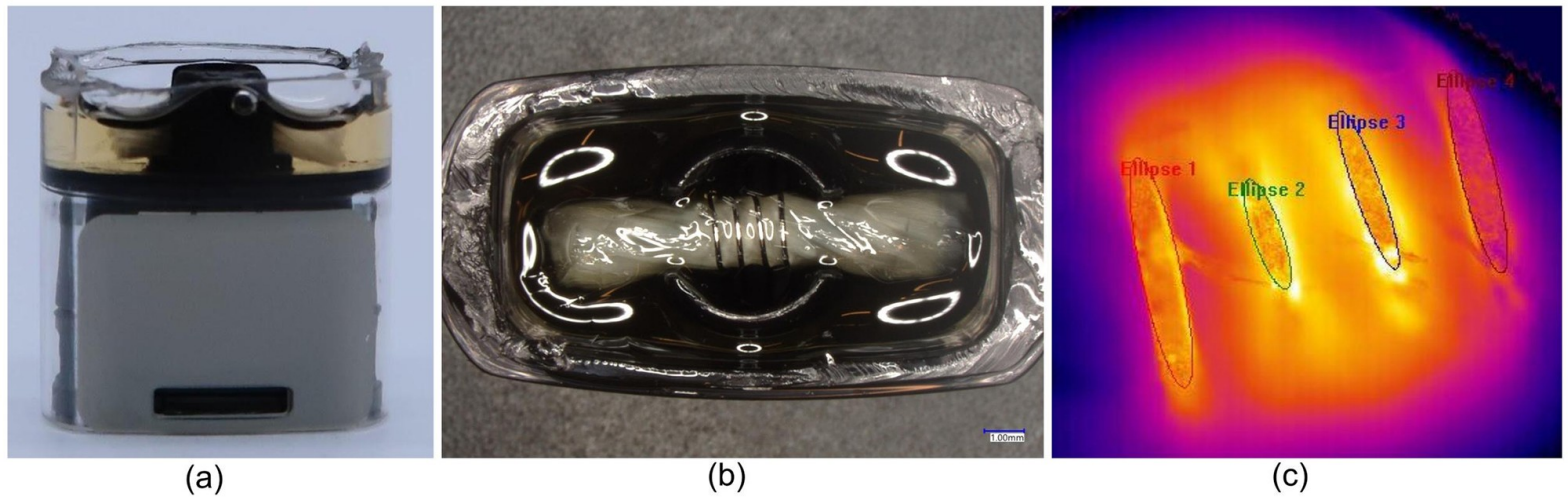
Thermography of the PAX^®^ Era^™^ pod temperature-controlled system (TC). (A) PAX^®^ Era^™^ pod cut, filled with cannabis extract, and prepped for thermography. (B) Optical microscopy image of a top-down view of a cut and prepped PAX^®^ Era^™^ pod filled with cannabis extract (not painted black for clarity). (C) IR thermography image of a PAX^®^ Era^™^ pod coil. Four elliptical regions of interest (ROIs) were drawn using the ResearchIR software specifying the area of the video that temperature was measured.

Cartridges and pods were cut open to expose the coil for temperature measurements and then lightly spray painted black to increase their emissivity. The cartridge or pod was filled halfway with the cannabis extract. Filled cartridges and pods were placed in a 50 °C oven for 8–10 minutes to allow the cannabis extract to fully saturate the ceramic core or silica wick prior to being puffed (Fig 2). After saturation, the coils were primed and stabilized by subjecting the saturated coils to 10 repeated puffs on the highest voltage or temperature setting for the respective devices. The VV510 was primed at 4.0V, the SV510 was primed at its sole 3.2V setting, and the TC was primed at 420 °C. Thermography was performed using fully charged batteries and primed cartridges spaced at least two minutes between consecutive puffs to allow wick and coil resaturation and to prevent puffing on a desaturated coil (i.e., “dry hits”).

**Fig 3:**
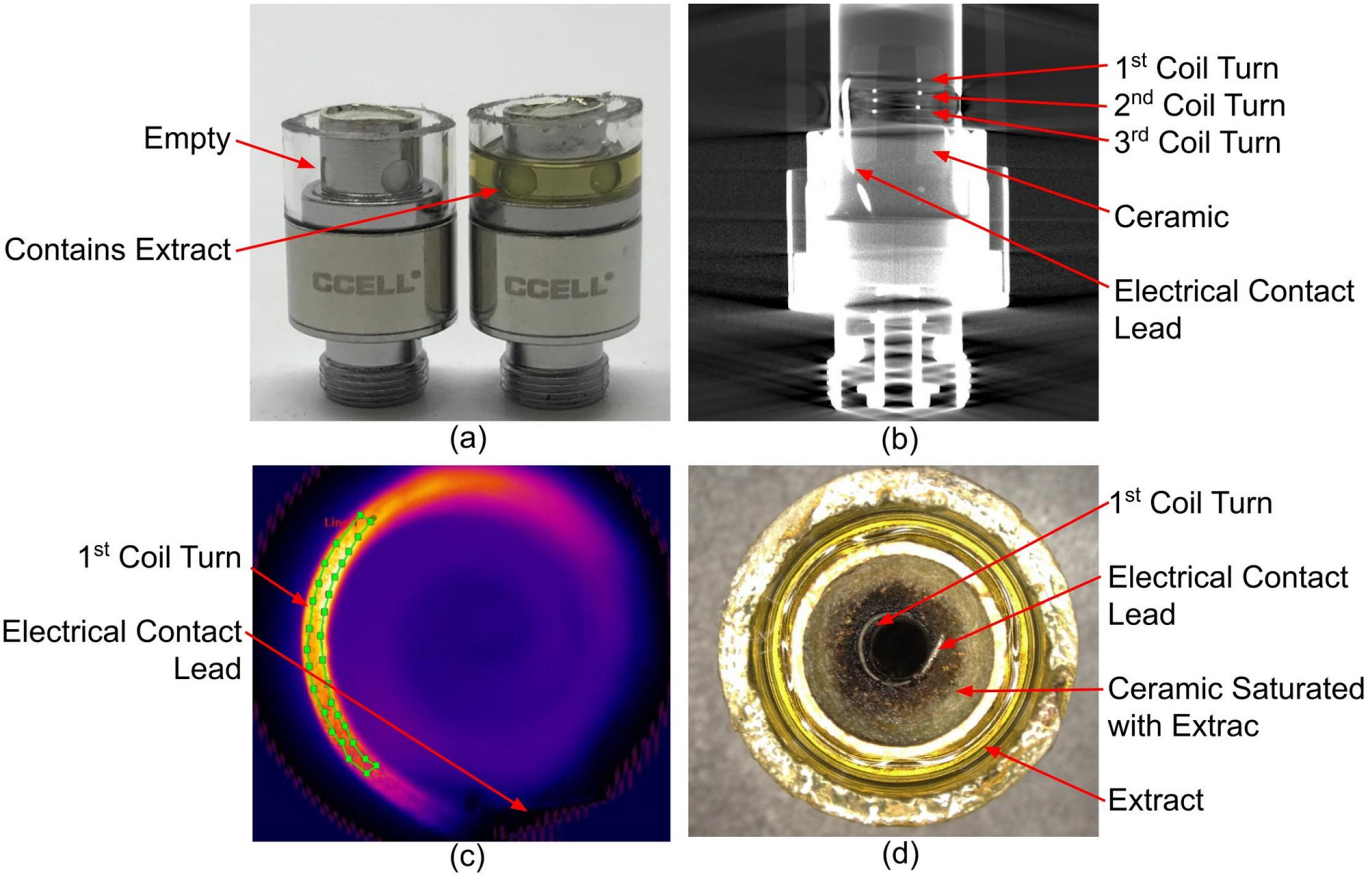
510 cartridge thermography for the VV510 and SV510 systems. (A) Side view cartridge cut down, filled and prepped for thermography. (B) X-ray Computed Tomography image of the cartridge’s side profile with three visible coil turns. (C) IR thermography image of the first coil turn that has been exposed from the ceramic. The green curved box is the ROI drawn to collect coil temperature measurements. (D) Top view optical microscopy image of a cut and prepared cartridge (post saturation and priming) from the top looking down into the atomizer.

The CCELL^®^ heating coil windings are embedded within a cylindrical ceramic wick and thus are more difficult to directly visualize. To access the CCELL^®^ heating coils, the cartridges were cut in half leaving enough of the tank remaining so that cannabis extract can be present to saturate the wick and coil during puff testing (Fig 3). Additionally, the top portion of the ceramic wick was removed through abrasion to allow a direct line-of-sight on the top wire turn of the heating coil. Removing the top portion of the cartridge is not expected to impact the CCELL^®^ coil temperatures because the features that impact coil temperature remain unchanged (i.e., the wick and coil stay saturated with extract, air path diameter is unchanged, and the air flow rate is maintained at 2 liters per minute (L/min)).

### Coil temperature measurement using IR thermography

**Fig 4:**
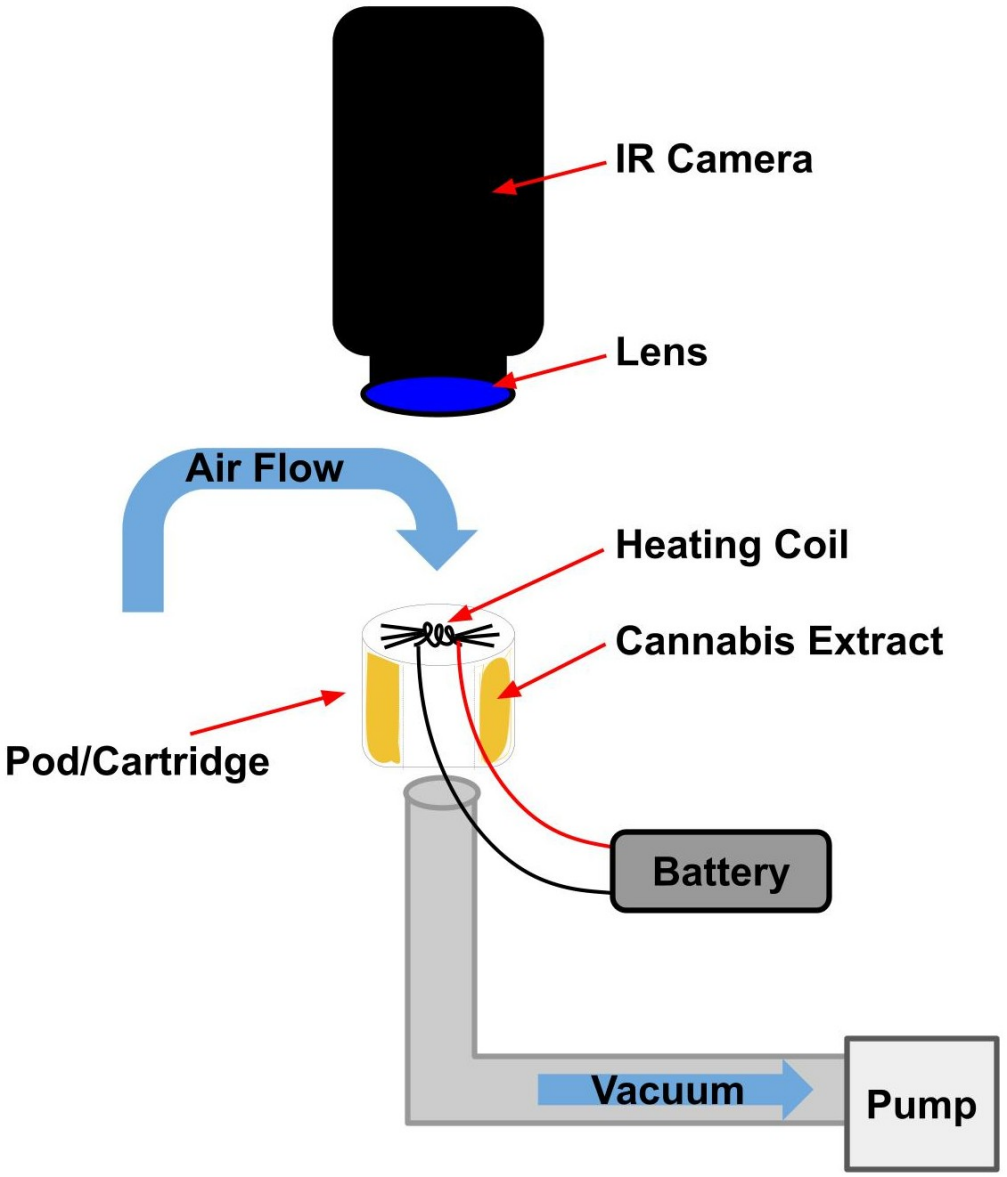
Thermography setup. Not drawn to scale; diagram is for illustration purposes only.

In order to measure the heating coil temperatures that are representative of actual human usage of vaporization systems, the thermography set-up and fixtures were designed specifically to: 1) situate the pod relative to the camera so as to give a direct line-of-sight on the heating coils, while, 2) the wick and coil were fully saturated with cannabis extract, and 3) puffed with 2 L/min of air flow moving past the coil with a square wave on/off shape for the full duration allowed by the device (Table 1, Fig 4, S1-S3 Figs in S1 File). The flow rate of 2 L/min is representative of a typical puff [21] however the air flow direction across the coils was reverse to that in actual use. Reverse air flow direction was used to pull the vapor away from the camera to not obstruct view of the coil. While changes to the air flow rate can impact the coil temperature, the flow rate direction is not expected to impact the temperature of the heating coil. For example, increasing the air flow rate that is moving past the heating coil will pull greater heat away from the coil leading to a reduced coil temperature. Changing the air flow direction—while keeping the flow rate constant—is not expected to impact the coil temperature because the same amount of air is flowing past the coil regardless of the flow direction.

The vacuum was also essential in activating the SV510 and VV510 batteries as they are actuated by consumer inhalation. The fixtures allowed the pods and cartridges to remain upright, so the cannabis extract remained at an even level around all sides of the wick and coil. When recording, each device fixture was placed under the lens of the camera on an adjustable platform to facilitate imaging (Fig 4).

The TC system was controlled using PAX^®^’s internal computer-based command line input (CLI) to set the coil temperature as well as turn the coil on and off. While the PAX^®^ device temperatures can be controlled by the consumer at single degree increments through the PAX App, CLI was preferred for these experiments because real-time streaming performance data output including coil resistance, voltage input, and other parameters of the puff event can be gathered.

The VV510 and SV510 could not be controlled by CLI access and were activated using the 2 L/min flow rate vacuum pressure to actuate heating. During a puff taken from one of the voltage-controlled devices, the vacuum activated the device by simulating a puff, which subsequently powered the heating coil, and a thermography video was recorded for further analysis. The cartridge was allowed to rest for at least 2 minutes between puffs to resaturate the wick and coil prior to successive puff events and to prevent “dry hits.”

All coil temperature measurements were collected using a model A8303sc FLIR Systems Inc. brand IR thermography camera with an attached 4X lens and Neutral Density #1 filter. The camera and lens combination were calibrated by the manufacturer from 200 to 600 °C based on pre-defined factory calibration ranges available, which encompasses the coil temperatures in the ENDS literature and the TC temperature setting range, and cannabinoid boiling temperatures. The calibration is accurate to ± 2 °C or 2%, whichever is greater, and all measurements were performed less than one year after factory calibration. The camera utilized a high-definition format of 1280x720 pixel array, and a recording rate was 60 frames per second. Using the 4X microscope lens, spot sizes can be measured down to 5 microns/pixel. Coil temperature measurements were performed using ResearchIR software (version: ResearchIR 4). Puffs were recorded on each device to measure the temperatures reached by the coil during the puff. The devices were puffed for the maximum duration allowed by the device. The VV510 device allowed four second puffs at the high voltage setting and eight second puffs at the low voltage setting. The SV510 device allowed 10 second puffs while the TC system allowed 15 second puffs.

To gather a complete and accurate representation of the coil temperature throughout a puff, a recording of the coil during the entire puff event was collected as follows:

1. For VV510 and SV510, the camera began recording briefly before the puff was initiated to ensure visualization of the coil as it heated up. Then the vacuum was switched on, which activated the battery. Once the puff event was finished, the camera continued to record for at least 5 additional seconds. This was to ensure that the coil cool down was also recorded.
2. Similarly, for the TC system, the camera started recording before the puff was initiated. The vacuum was activated before the camera began recording, and the puff was started through a CLI command. Once the puff was finished, the camera recorded for at least 5 additional seconds to visualize the coil cool down.

### IR video analysis

Coil temperature quantification was performed using ResearchIR software. Regions of interest (ROIs) were drawn in the thermography video on the sections of coil that were in focus (Fig 2B) and the software calculated the minimum, maximum, and mean temperatures within the ROI. As shown in Fig 2B, all four turns of the TC heating coil can be observed simultaneously. The reported coil temperatures were generated by calculating the average ROI mean temperatures of the four ellipses. Three PAX^®^ Era^™^ pods were tested for this analysis; therefore 3 different coils were measured. The same three CCELL^®^ 510 cartridges were used for both the VV510 and SV510 devices. For the VV510 and TC systems, coil temperatures were recorded for the highest and lowest settings on the devices. Overall, three cartridges were analyzed on each device and were puffed at the full puff length allowed by the respective devices, which is when the device powers itself off.

The heating coil temperatures of the 510 voltage-controlled systems (VV510 and SV510) were collected similarly to the TC system, although the ROIs were altered slightly to fit the coil shape (Fig 3C). Since only the first turn of the 510 coil was visible from a top-down view, the ROI was drawn as a curved line along the coil carefully avoiding the electrical contact posts. Measurements along the line ROI was used to calculate the average temperature of that coil turn. The temperature of this outer coil turn was used to estimate the average temperature of the whole coil.

## Results

### Objective 1: IR thermography as a method to measure heating coil temperature during a simulated puff event

This study describes a thermal imaging method to measure cannabis extract vaporizer heating coil temperatures under simulated human usage conditions. To ensure accurate coil temperature measurements of simulated human usage, numerous conditions were required. Namely, the wick and coil were fully saturated with cannabis extract during the entire puff event, air flow past the coils was maintained at 2 L/min for the full duration allowed by the device, and the IR camera was factory calibrated from 200 to 600 °C.

Measured coil temperatures were almost entirely within the calibration range of the IR camera. The detector showed minimal measurements above the 600 °C defined calibration range for one of three replicates. Specifically during one of the high setting (4.0V) VV510 replicates, the measured temperature exceeded 600 °C for 700 msec. When the coil temperature is only slightly outside of the calibration range for a very limited amount of time the effect on the overall reported average temperatures would be minimal. The average heating coil temperatures of three different vaporization systems measured during a simulated puff event using IR thermography ranged from 238–642 °C. A wide temperature range was observed between the systems analyzed with only the TC device capable of holding the lower temperatures (<400 °C) for the duration of the puff allowed by the device.

### Objective 2: Comparison of heating coil temperature in temperature-controlled and voltage-controlled vaporization systems

**Fig 5:**
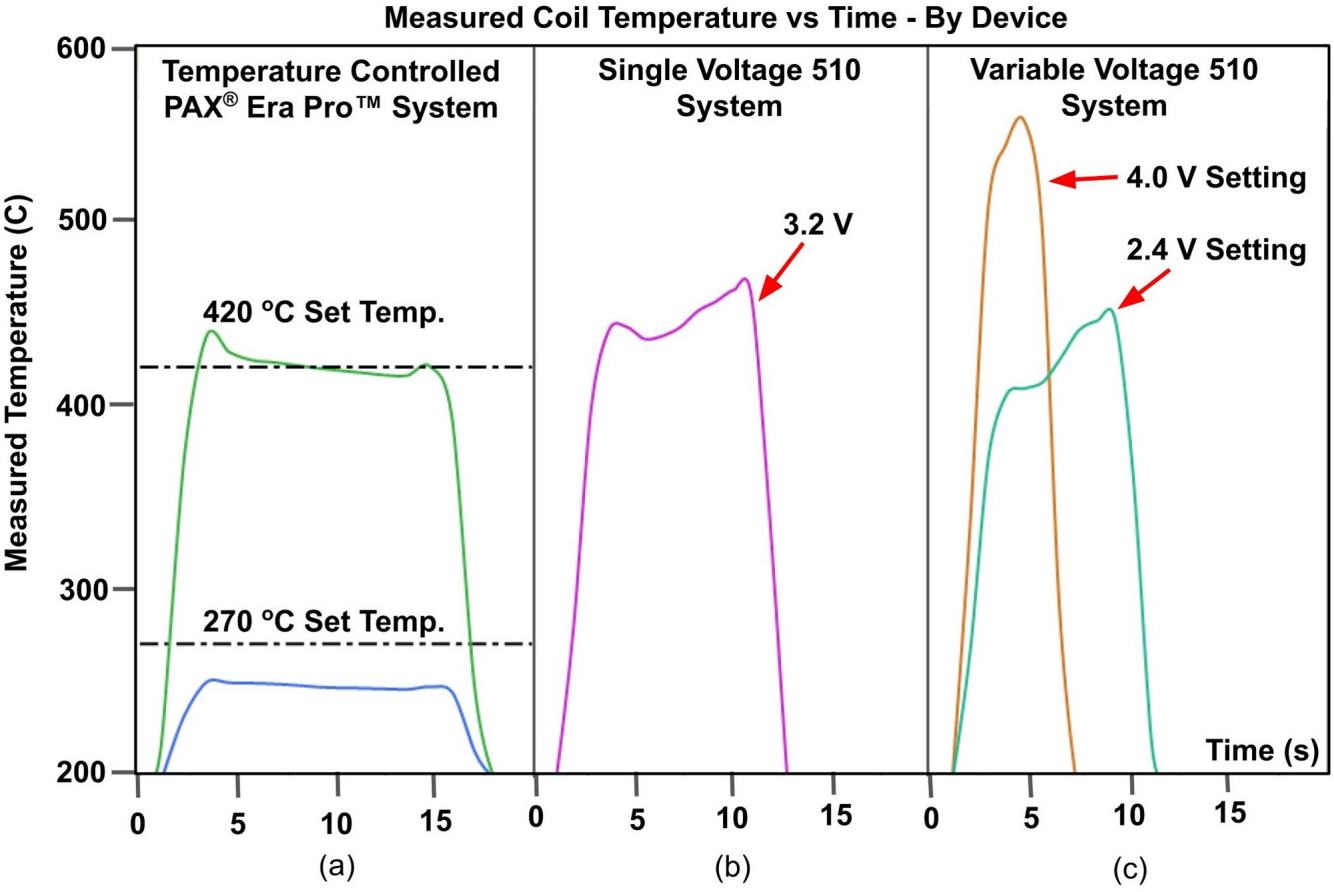
Average measured coil temperature versus time. (A) PAX^®^ Era Pro^™^ pod temperature-controlled system (TC). (B) single voltage device paired with a 510 cartridge (SV510). (C) variable voltage device paired with a 510 cartridge (VV510).

A direct comparison of IR thermography temporal plots was made between coil temperatures observed for the TC, VV510, and SV510 systems (Fig 5 and S4 Fig in S1 File). At the start of a puff, the measured coil temperature rapidly rises until the set temperature or set voltage is reached. The coil temperature then holds constant at the set temperature or voltage throughout the duration of the puff and this is seen as a plateau in the temporal plot. At the end of the puff event, the coil is turned off and the measured temperature rapidly falls. Average heating coil temperatures of the 510 voltage-controlled systems’ low voltage settings (or only setting for SV510) were similar to the TC system’s highest temperature setting. Overall, the TC system had the capability to operate at much lower temperatures than the 510 voltage-controlled systems analyzed in this study. The SV510 system generated average coil temperatures (± standard deviation) of 450 ± 60.8 °C. The highest setting on the VV510 (4.0V) generated average temperatures of 543 ± 95.9 °C whereas the TC system generated average temperatures of 420 ± 9.5 °C at the highest temperature setting. The TC system’s lowest setting generated an average temperature of 246 ± 5.1 °C and even lower temperature settings (as low as 220 °C) are available through the mobile or web application. In addition, the accuracy of the TC system’s coil temperature was within 10% of the set temperature and when temperatures deviated from the set temperature, they tended to be lower.

The voltage setting during a puff did not have a direct linear correlation to the measured temperature. The SV510 system had a labeled output of 3.2V with an average temperature of 450 ± 60.8 °C which was similar to the VV510 setting of 2.4V which had an average temperature of 443 ± 56.1 °C (S1 Table in S1 File). If voltage were the only contributor to temperature, the 3.2V SV510 would be predicted to be halfway between that of the VV510 device settings (443 and 543 °C) at approximately 493 °C. As such, other factors besides voltage must play a role in coil temperature. Coil temperatures within the VV510 did act as expected with higher voltage settings trending toward a higher temperature although there was variability in temperature within a voltage setting (S1 Table in S1 File).

## Discussion

### Thermography is a useful method for measuring temperature at the site of vaporization during a simulated puff in cannabis extract vaporizers

The TC system’s measured coil temperature was within 10% of the set temperature (S1 Table in S1 File) suggesting that IR thermography is an accurate way to measure heating coil temperature during vaporization. For voltage-controlled devices, more variability in temperature was observed than the TC system; however, based on the interchangeable nature of 510 cartridges and 510-threaded batteries this is expected. This method is preferable over other methods because a greater portion of the heating coil can be measured and temporally resolved which gives a more complete picture of the temperature during an entire puff than the use of thermocouples. This is important because the temperature can vary along the length of the heating coil and throughout a puff as shown in Fig 5.

A limitation of this work is that only a small sampling of the myriad 510 vaporization systems on the market were analyzed. The brands of these devices were randomly chosen within a broad price range and thus are believed to be representative of a large portion of those available on the market. All 510-compatible cartridges and devices are connected to one another using a universal mating thread design; therefore any manufacturer can produce a cartridge or device for the 510 ecosystem. Due to the wide variability in the 510 ecosystem with regard to materials used in construction, types of heating coils, and device voltage controls, we expect to see a widely varying range of coil temperatures and performance. In contrast, proprietary closed-loop temperature-controlled vaporization systems ensure consistent temperature control by design.

In this study, several modifications from an actual human puff event were required that could affect the temperatures measured during these simulated human puff events. Firstly, pods and cartridges had to be cut to expose the heating coil for thermography. The features that are expected to impact coil temperature remain unchanged thus this method would provide a good estimate of coil temperature. Secondly, for the voltage-controlled 510 devices only the top winding of the coil could be accessed for thermography. In general, resistively heated vaporization coils typically show greater temperatures at the center of the coil and lower temperatures away from the center and near the electrical contact posts. While the TC pod coil windings are oriented such that the temperature of each wire turn can be thermally imaged, this is not feasible in a 510 cartridge due to the orientation of the 510’s coil windings relative to the air path and because the coil is partially embedded in ceramic. As such, the coil turn that is closest to the mouthpiece of the 510 cartridges was thermally imaged. Since only the first turn of the CCELL coil was thermally imaged (Fig 3), we expect the reported CCELL coil temperatures to be a slight underestimate of the overall average coil temperature. The reason for this underestimate is because the center of a vaporization heating coil is typically hotter than the ends due to thermal conduction away from the coil and into the heat sinking electrical contact leads on either end [19]. Finally, the puff lengths differed between each system and were longer than most consumers are likely to draw from the device to determine worst case. This would unlikely change the average temperature of the TC system since the temperature remains relatively constant through the entire puff. Also, for the VV510 4.0V setting the allowed puff duration was already short (4 sec). A shorter puff duration might show lower average temperatures for the SV510 and VV510 2.4V setting based on the shapes of the curves (Fig 5). While this might reduce the risk for some consumers, there are still some consumers that will take longer puffs and use the high voltage settings.

### Heating coil temperature and implications for consumer risk

All vaporization systems analyzed in this report were below temperatures reached in a cigarette during a puff (850–920 °C) and below that of a cannabis “joint” which is hypothesized to burn hotter than tobacco cigarettes [22, 23]. Importantly, the temperature-controlled system was able to generate a wide range of temperatures that included much lower coil temperatures than either of the voltage-controlled 510 systems included in this study. Temperatures observed in this study were demonstrably higher than previously published values that found temperatures up to 400 °C [18, 19, 24, 25]. Temperatures are likely higher in the devices included in this study since they were designed for cannabinoid vaporization which require higher temperatures than nicotine. Previous studies examined ENDS designed for vaporization of nicotine-containing liquids which have a lower boiling point than cannabinoids. "Dry hits” from unsaturated wicks could also lead to higher temperature measurements [11, 19, 26] however this was unlikely to be a factor in the study as sufficient time was allowed for wick resaturation for each puff, and coils were closely monitored during these studies to prevent “dry hits.”

Published research on both cannabis and nicotine vaporization products has demonstrated that higher concentrations of undesirable and potentially toxic thermal degradation products are produced as temperature (or device power output) increases [7, 27–29]. These thermal degradants include aromatics (e.g., benzene, styrene), carbonyl compounds (e.g., formaldehyde, methacrolein), and others (e.g., polyaromatic hydrocarbons, carbon monoxide) that should be avoided due to the potential for toxicity. Unfortunately, many studies do not report coil temperatures and only report power output of the device, so it is unclear what implications these temperatures have for thermal degradants produced and consumer risk. Of course, concentration and identity of thermal degradants formed is dependent on substances present in the cannabis extract and coil temperature; however, there is often a steep increase in degradant concentration once the inflection point is reached thus consistent coil temperature is likely important for controlling health risk [2, 28, 29].

## Conclusions

We have found that thermography is a useful method for measuring temperature at the site of vaporization during a simulated puff in cannabis extract vaporizers. This method can be useful in further characterizing heating coil temperatures with different vaporization systems and to characterize how different puffing parameters can affect temperature. In addition, knowledge of heating coil temperatures may be useful in predicting potential thermal degradation products from cannabis extracts and additives.

The temperature-controlled system could operate at much lower coil temperatures than either of the voltage-controlled 510 systems included in this study. These lower temperatures could reduce the likelihood of harmful thermal degradation products and thus may reduce potential health risk to consumers from exposure when vaporizing cannabis extracts. There are few studies evaluating thermal degradation products formed during vaporization of cannabis extracts. Future work in our laboratory will examine thermal degradation products present in aerosol produced by these devices at different temperatures and voltage settings. Finally, these methods may be useful in public health harm reduction approaches for cannabis extract vaporization products.

## Supporting information

S1 File (DOCX)
