# Supplementary material for: Thermography of cannabis extract vaporization cartridge heating coils in temperature- and voltage-controlled systems during a simulated human puff
Source: PLoS One. 2022 Jan 26;17(1):e0262265. doi: 10.1371/journal.pone.0262265 (PMC8791474; doi:10.1371/journal.pone.0262265)
Supplement: S1 File — (DOCX) [file pone.0262265.s001.docx]

# SUPPORTING INFORMATION

## **Coil and IR thermography preparation**

All 510 cartridges (CCELL® M6T10 - size 1-gram) were cut horizontally across the width of the tank with a Dremel tool (SR Foredom with 1.5” blade). Cartridges were cut down to about ⅓ of the original size to expose the coil and conduct temperature measurements. The top ⅔ of the plastic tank was cut away and discarded. The main, thin portion of the metal chimney was also cut off where it met the metal ceramic housing. This left enough of the plastic tank to fill with cannabis extract and saturate the ceramic wick and coil. An Exacto™ blade was used to smooth the edges of the cartridge, and air was used to remove any debris from cutting. PAX® Era™ pod used in this study were also cut down. The mouthpiece and wick housing were removed, and the coil housing was pushed out of the plastic tank. The plastic tank was cut in half, where the bottom of the mouthpiece sits on the tank, using scissors.

All exposed coils used in this study were painted with high temperature black spray paint (Krylon® Specialty High Heat Max). After a 510 cartridge was cut, the coil needed to be exposed since the coil was fully embedded in the ceramic. The top portion of the ceramic was carefully scraped off until the first turn of the coil was visible. Coils were fully coated with the black paint and left to dry overnight. PAX® Era™ pods did not require further alteration to visualize the coils since the coils were visible after just cutting the plastic tank. Painting the coils black increased their emissivity and resulted in better thermal imaging. A needle and syringe were used to fill the 510 cartridges (BD PrecisionGlide Needle 20G x 1 attached to a 10mL syringe). Filling was done in front of a heat gun to decrease the viscosity of the cannabis extract and expedite moving the extract through the syringe needle. The cut cartridge tank was filled to a level just above the inlet openings on the bottom of the metal chimney. By covering the inlet openings of the metal chimney with cannabis extract, we ensured that the extract was in contact with the ceramic wick and was fully saturated during testing. PAX® Era™ Pods were filled using just the 10mL syringe without a needle containing the same cannabis extract because the PAX® Era™ pod’s tank opening was much larger than the 510 cartridges’ it did not require a small needle.

To focus the IR camera on the coils in the cartridges while they were attached to the batteries, fixtures needed to be created for each device. The PAX® Era™ device fixture allowed for a cut and prepped PAX® Era™ pod to be electrically connected to the device, and computer commands were used to change temperature and start the puffs (S1 Fig). The rubber adapter around the PAX® Era™ pod was used to ensure proper sealing and air flow. All device fixtures allowed for simulation of human consumer conditions during a puff. For both the SV510 and VV510 systems, the top electrical contact where the cartridge screws into the battery was removed, with the internal wiring to the rest of the battery kept intact (S2 and S3 Figs). This allowed for vacuum lines to be connected separately to the vaporization device (to turn it on) and to the cartridge (to pull the air over the coils). These fixtures were attached to a movable IR camera. The IR camera was attached to the upper portion of this stage and could be raised or lowered to adjust focusing.

**S1 Fig. PAX® Era™ system fixture.** The IR camera is pointed down towards the pod and has direct line-of-sight on the heating coils. The device used to power the heating coils is shown on the right and the vacuum line is seen on the left.

**
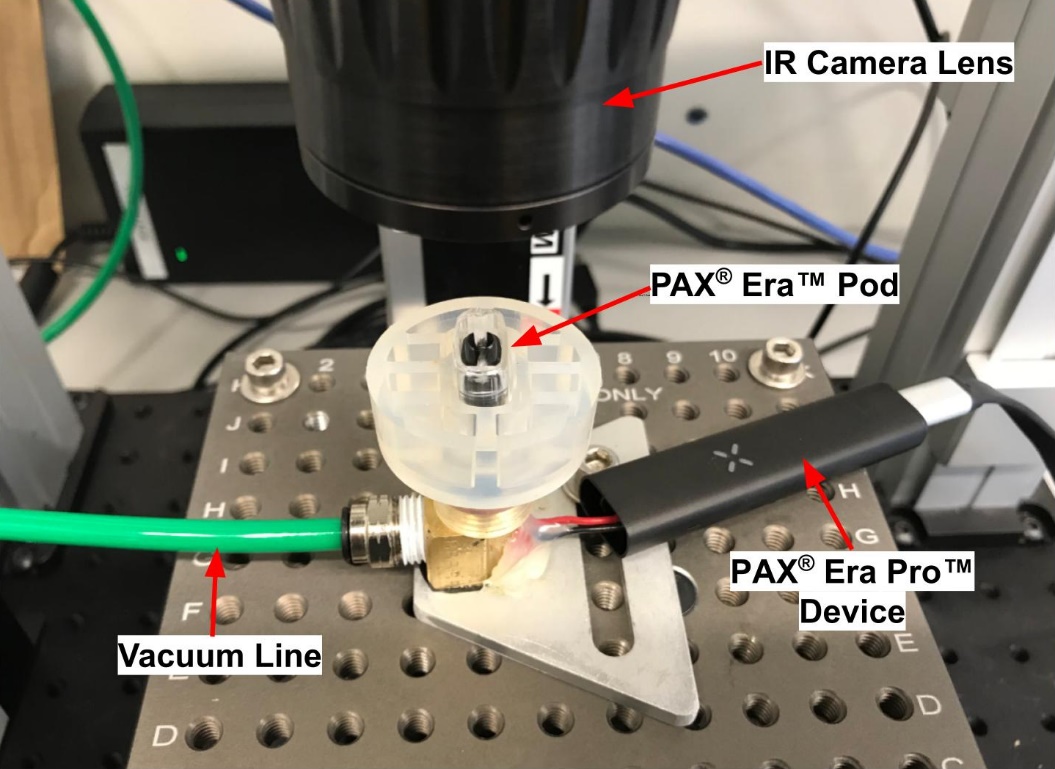
**

**S2 Fig. Single voltage battery (SV510) fixture.** The IR camera is pointed down towards the cartridge and has direct line-of-sight on the heating coils. The device used to power the heating coils is shown on the right and the vacuum lines are seen on the left.

**
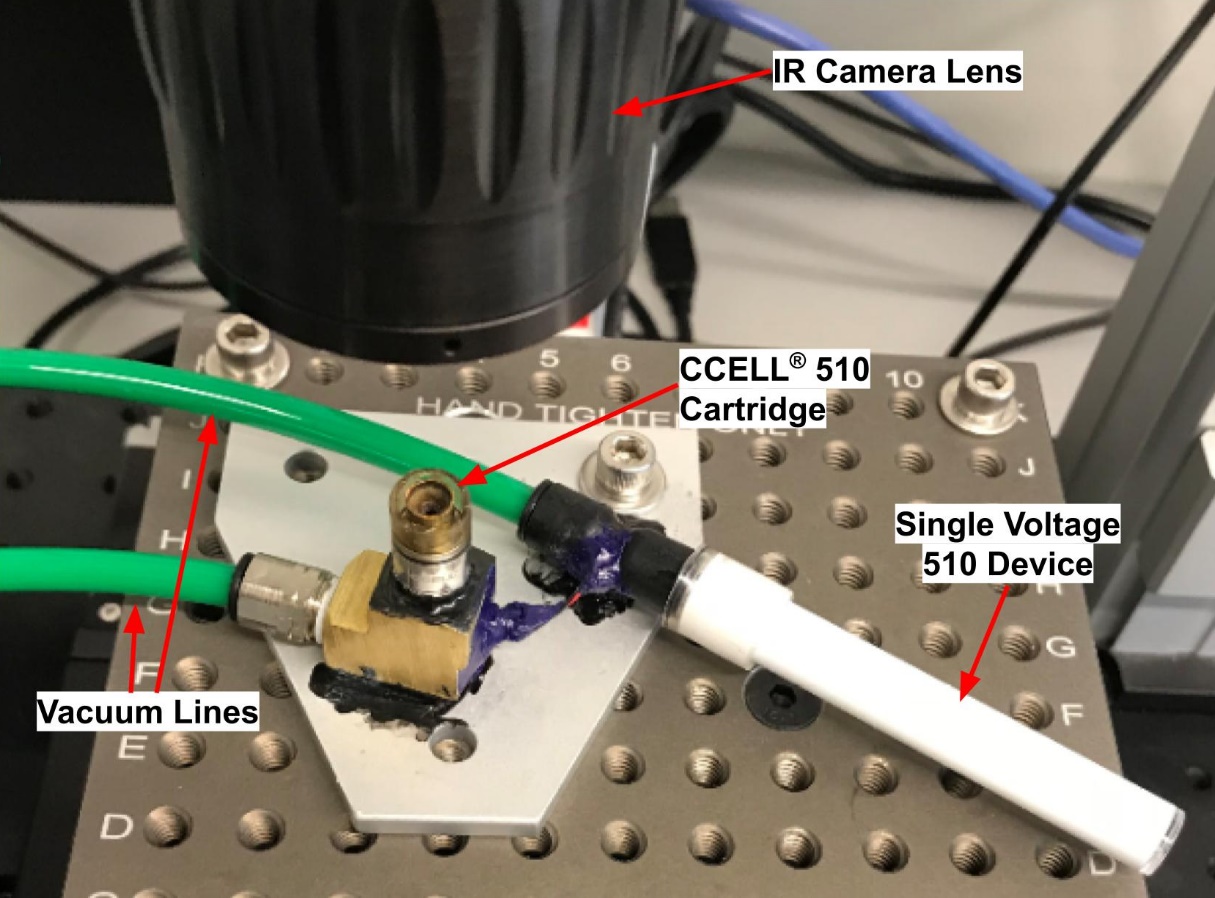
**

**S3 Fig. Variable voltage battery (VV510) fixture.** The IR camera is pointed down towards the cartridge and has direct line-of-sight on the heating coils. The device used to power the heating coils is shown on the right and the vacuum lines are seen on the left.


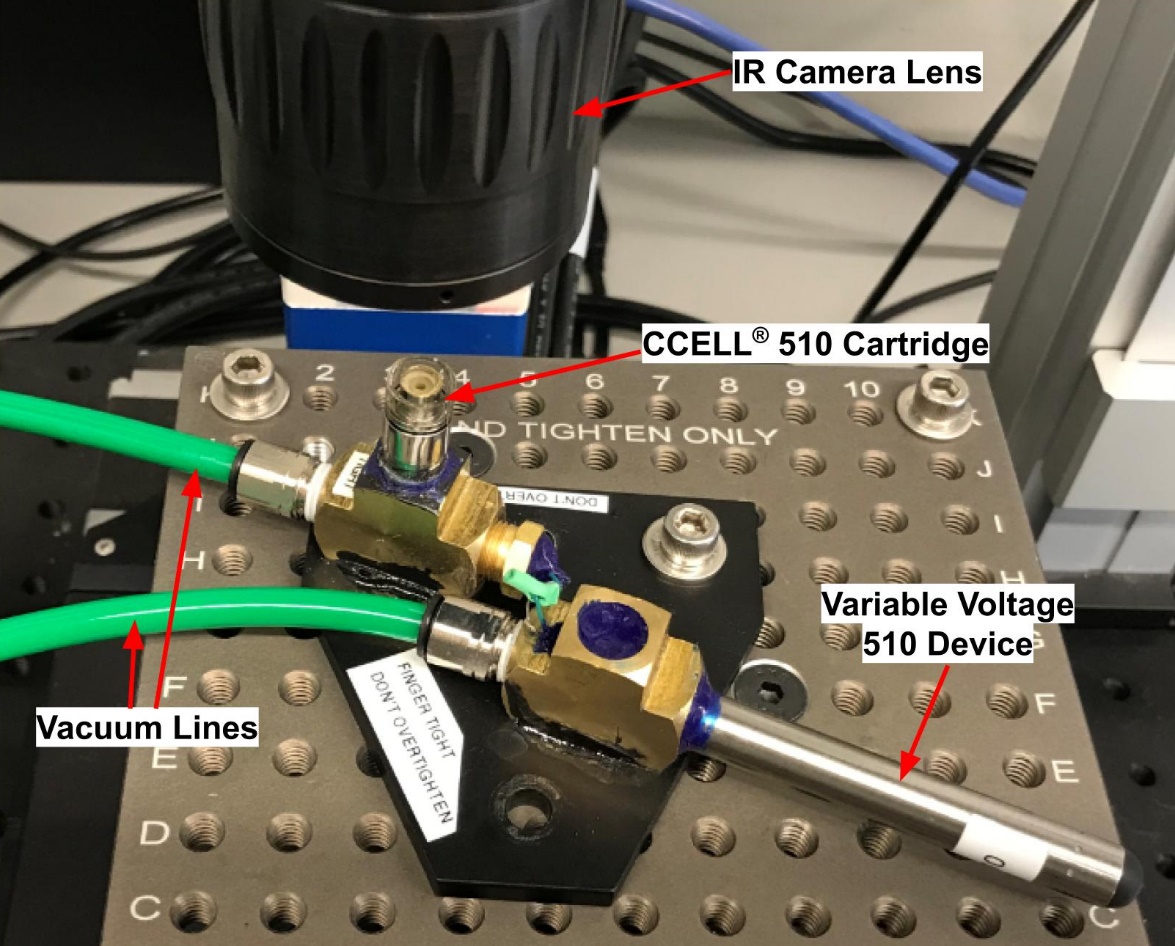


## **Raw thermography data for temperature- and voltage-controlled systems**

Raw thermography data for each device and the three pods tested on each device are shown in S1 Table. Coil temperature vs time data are also shown below in S4 Fig for each replicate in each system.

| S1 Table. Vaporization System Coil Temperature Measurements Performed in Triplicate   \| **System** \| **Device setting** \| **Replicate** \| **Measured temperature of ROI (^o^C)** \| \| \| **Trial statistics** \| \| \| --- \| --- \| --- \| --- \| --- \| --- \| --- \| --- \| \| **Min.** \| **Max.** \| **Mean** \| **Mean ± s.d.** \| **Range** \| \| Temperature-controlled system \| Lowest  temp.  (270 ^o^C) \| 1 \| 248 \| 256 \| 252 \| 246 ± 5.1 \| 238-256 \| \| 2 \| 242 \| 250 \| 245 \| \| 3 \| 238 \| 247 \| 242 \| \| Highest  temp.  (420 ^o^C) \| 1 \| 400 \| 439 \| 410 \| 420 ± 9.5 \| 400-462 \| \| 2 \| 419 \| 462 \| 429 \| \| 3 \| 409 \| 453 \| 421 \| \| Variable voltage system \| Highest Voltage  (4.0 V) \| 1 \| 549 \| 597 \| 594 \| 543 ± 95.9 \| 398-648 \| \| 2 \| 398 \| 451 \| 432 \| \| 3 \| 567 \| 648 \| 602 \| \| Lowest voltage  (2.4 V) \| 1 \| 396 \| 496 \| 478 \| 443 ± 56.1 \| 326-496 \| \| 2 \| 326 \| 385 \| 378 \| \| 3 \| 435 \| 496 \| 472 \| \| Single voltage system \| Single voltage setting  (3.2V) \| 1 \| 354 \| 418 \| 410 \| 450 ± 60.8 \| 354-526 \| \| 2 \| 375 \| 442 \| 420 \| \| 3 \| 510 \| 526 \| 520 \| |
| --- | --- | --- | --- | --- | --- | --- | --- | --- | --- | --- | --- | --- | --- | --- | --- | --- | --- | --- | --- | --- | --- | --- | --- | --- | --- | --- | --- | --- | --- | --- | --- | --- | --- | --- | --- | --- | --- | --- | --- | --- | --- | --- | --- | --- | --- | --- | --- | --- | --- | --- | --- | --- | --- | --- | --- | --- | --- | --- | --- | --- | --- | --- | --- | --- | --- | --- | --- | --- | --- | --- | --- | --- | --- | --- | --- | --- | --- | --- | --- | --- | --- | --- | --- | --- | --- | --- | --- | --- | --- | --- | --- |
| s.d., standard deviation |

**S4 Fig. Individual puffs per pod on each device.** Individual measured coil temperature over time. (A) Temperature-controlled PAX® Era™ pod system (TC). (B) Single voltage device paired with a 510 cartridge (SV510). (C) Variable voltage device paired with a 510 cartridge (VV510).


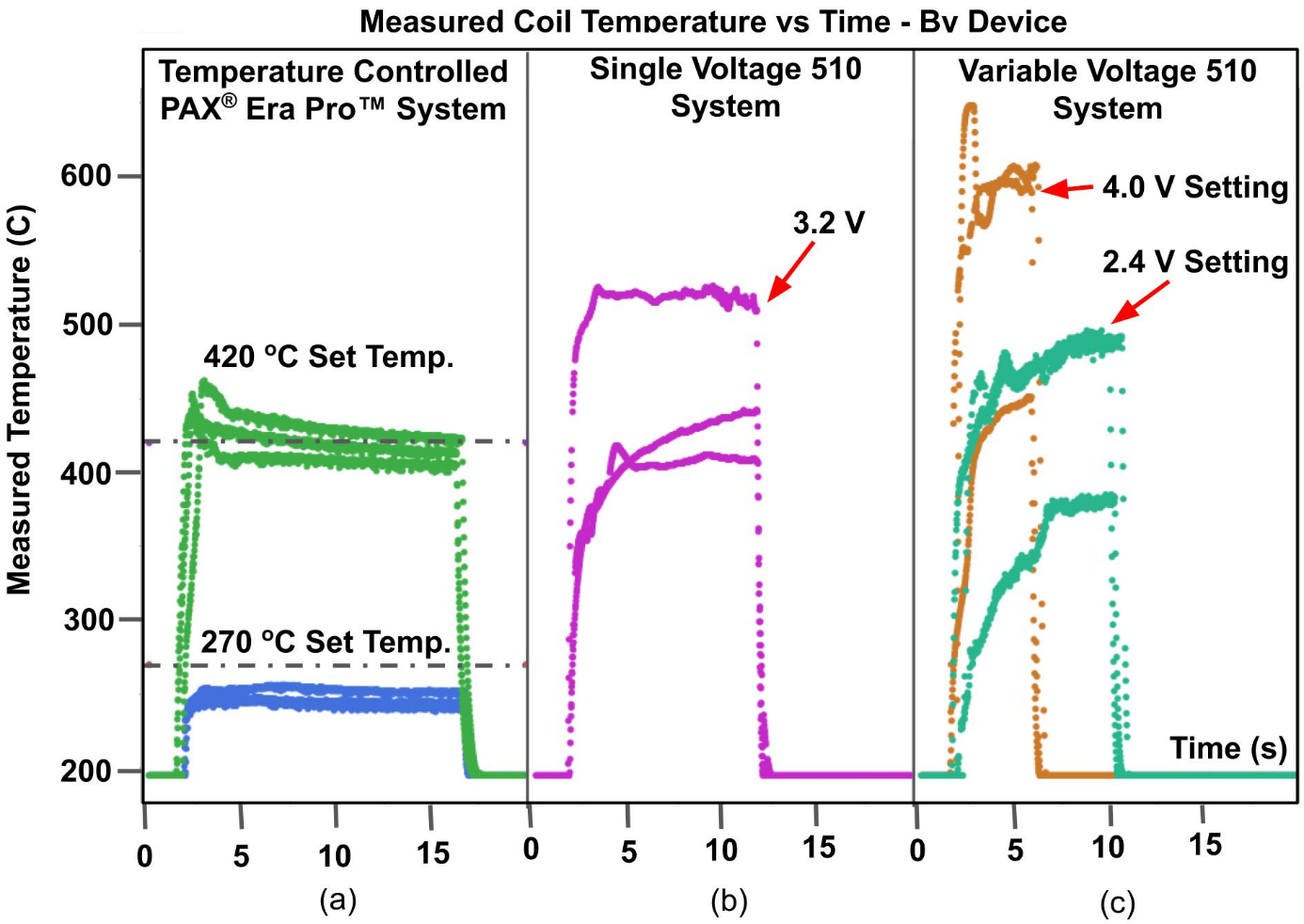


## **Comprehensive discussion around S4 Fig**

A direct comparison of IR thermography temporal plots was made between coil temperatures observed for the TC, VV510, and SV510 systems (Fig 5 and S4 Fig). These temporal plots are a graphical representation of the mean measured coil temperatures at a particular puff time. At the start of a puff, the measured coil temperature rapidly rises until the set temperature or set voltage is reached. The coil temperature then holds constant at the set temperature or voltage throughout the duration of the puff and this is seen as a plateau in the temporal plot. At the end of the puff event, the coil is turned off and the measured temperature rapidly falls. Three pods were analyzed on the TC system and three carts were analyzed on both the VV510 and SV510 systems. Temporal plots for each analysis are presented in the supplemental information (S4 Fig) and the average of these three runs is presented in Fig 5.

S4 Fig, Inset A presents the overlaid temporal plots for three pods puffed on the TC system. The green lines correspond to the measured coil temperatures that were observed for three different pods when the device was set to 420 ^o^C. The blue lines correspond to the measured coil temperatures that were observed for the same three pods when the device was set to 270 ^o^C. A small amount of variability is observed during replicate measurements possibly due to slight differences in coil orientation, drawn ROI differences and differing levels of extract saturation of the wick and coil. The PAX® Era Pro™ device (firmware version 4.2.3) has a maximum allowed puff length of 15 seconds so the device turns the coil off after 15 seconds.

S4 Fig, Inset B presents the overlaid temporal plots for three cartridges puffed on the SV510 system. The purple lines correspond to the measured coil temperatures that were observed for three different cartridges at the sole 3.2V setting of the SV510 system. Variability is again observed during replicate measurements possibly due to slight differences in coil orientation, drawn ROI differences and differing levels of extract saturation of the wick and coil. The SV510 system has a maximum allowed puff length of 10 seconds so the coil is seen to turn off after 10 seconds.

S4 Fig, Inset C presents the overlaid temporal plots for three cartridges puffed on the VV510 system. The brown line corresponds to the measured coil temperatures that were observed for three different cartridges when the device was set to the highest setting of 4.0V. The teal line corresponds to the measured coil temperatures that were observed for three different cartridges when the device was set to the lowest setting of 2.4V.
